# Effect of Breast Milk Oral Care on Mechanically Ventilated Preterm Infants: A Systematic Review and Meta-Analysis of Randomized Controlled Trials

**DOI:** 10.3389/fped.2022.899193

**Published:** 2022-07-07

**Authors:** Meiling Cai, Lingyu Lin, Yanchun Peng, Liangwan Chen, Yanjuan Lin

**Affiliations:** ^1^Department of Cardiac Surgery, Union Hospital, Fujian Medical University, Fuzhou, China; ^2^Department of Nursing, Union Hospital, Fujian Medical University, Fuzhou, China; ^3^School of Nursing, Fujian Medical University, Fuzhou, China

**Keywords:** breast milk, oral care, mechanical ventilation, preterm infants, meta-analysis

## Abstract

**Background:**

The benefits of breast milk oral care in mechanically ventilated preterm infants remain controversial. This study aimed to systematically review the evidence on the benefits of breast milk oral care in mechanically ventilated preterm infants.

**Methods:**

The randomized controlled trials of breast milk oral care for mechanically ventilated preterm infants were searched in EMBASE, PubMed, Cochrane Library, Web of Science, WANFANG Date and China National Knowledge Infrastructure databases. The retrieval language was limited to Chinese and English, and the final search was conducted until March 2022. Outcome measures included ventilator-associated pneumonia (VAP), mechanical ventilation time (MVT), length of stay (LOS), necrotizing enterocolitis (NEC), late-onset sepsis, mortality during hospitalization, time of full intestinal feeding and time of full oral feeding. Two researchers independently screened the literature, extracted the data, and conducted the literature quality assessment. Meta-analysis was mainly performed using RevMan 5.3.

**Results:**

Eight articles involving 1,046 preterm infants were included. Our meta-analysis showed that compared with the control group, breast milk oral care could reduce the incidence of VAP [*RR* = 0.41, 95% *CI* (0.23, 0.75), *P* = 0.003] and NEC [*RR* = 0.54, 95% *CI* (0.30, 0.95), *P* = 0.03], and shorten the MVT [*MD* = −0.45, 95% *CI* (−0.73, −0.18), *P* = 0.001] and LOS [*MD* = −5.74, 95% *CI* (−10.39, −1.10), *P* = 0.02]. There were no significant differences in the mortality during hospitalization [*RR* = 0.94, 95% *CI* (0.67, 1.33), *P* = 0.74], the incidence of late-onset sepsis [*RR* = 0.79, 95% *CI* (0.40, 1.59), *P* = 0.51], the time of full intestinal feeding [*MD* = −2.42, 95% *CI* (−5.37, 0.52), *P* = 0.11] and the time of full oral feeding [*MD* = −3.40, 95% *CI* (−10.70, 3.91), *P* = 0.36] between the two groups.

**Conclusions:**

Oral care of breast milk can reduce the incidence of VAP and NEC, shorten MVT and LOS in mechanically ventilated preterm infants. However, due to the quality and quantity limitations of the included studies, larger sample size and more strictly designed clinical trials are still needed in the future to further confirm the findings of this study.

## Introduction

Preterm birth is defined as being born before 37 weeks, and more than 41,000 babies worldwide are born before this gestational age every day (1). Preterm infants have immature lungs, lack surfactant, and immature respiratory control mechanisms, and mechanical ventilation (MV) is often required, which plays a vital role in reducing the early mortality of this population (2, 3). However, the establishment of artificial airway destroys the normal protective mechanism of respiratory tract in preterm infants, and the aspiration of oropharyngeal pathogenic bacteria is easy to cause ventilator-associated pneumonia (VAP) (4). Several studies have shown that strict and effective oral care can prevent oropharyngeal bacterial colonization and reduce the incidence of VAP (5–7). Therefore, oral care for mechanically ventilated preterm infants is particularly important to the prevention of VAP.

Breast milk is rich in immune active factors, which may be absorbed by the oropharyngeal mucosa of preterm infants, and can effectively inhibit the activity of oropharyngeal pathogens (8). Studies have shown that the use of breast milk as oral care solution is safe and effective, conducive to the rehabilitation of preterm infants, and can reduce the length of stay (LOS) (9). Recently, a meta-analysis published by Ma et al. (6) showed that oral administration of colostrum had a positive effect on reducing the incidence of VAP and necrotizing enterocolitis (NEC) in preterm infants and shortening the time of full intestinal feeding. However, this meta-analysis is mainly aimed at preterm infants (whether with or without MV), and the evidence for the effects of breast milk oral care (BMOC) on mechanically ventilated preterm infants remains insufficient. In addition, there is still controversy about the impact of BMOC on mechanically ventilated preterm infants, such as mechanical ventilation time (MVT) and LOS (6, 9–11). Therefore, this study focused on mechanically ventilated preterm infants and explored the impact of BMOC intervention on mechanically ventilated preterm infants through systematic evaluation and meta-analysis, so as to provide the scientific basis for clinical nursing.

## Methods

This systematic review with meta-analysis was conducted based on Preferred Reporting Items for Systematic Reviews and Meta-Analyses (PRISMA) guidelines (Supplementary File 1). The research protocol was registered and updated with PROSPERO under the registration number CRD42021273155.

### Literature Search

The databases, including Embase, PubMed, Cochrane Library, Web-of-Science, WANFANG Data and China National Knowledge Infrastructure (CNKI) databases were systematically searched up to March 2022, with Chinese and English language restrictions. At the same time, the references in the included literature and the articles quoting the included literature were traced to supplement and obtained the relevant literature. Medical subject words (MESH) and free words were used for retrieval, and the search words included: “Milk, Human,” “Colostrum,” “Intubation, Intratracheal,” “Ventilators, Negative Pressure,” “Ventilators, Mechanical,” “Child,” “Infant, Newborn,” “Infant, Premature,” and “Oral Hygiene.” We adjusted the search strategy and the full search strategy was presented in a supplemental document (Supplementary File 2).

### Study Selection

Endnote software was used for literature management and screening of duplicate studies. Qualified studies were independently identified and cross-checked by two researchers according to the inclusion and exclusion criteria below, and consulted by a third reviewer when necessary.

#### Inclusion Criteria

1) Population (P): Mechanically ventilated preterm infants. To improve the homogeneity and comparability of the study, we adjusted the study population from mechanically ventilated infants to mechanically ventilated preterm infants.
2) Intervention (I): Use BMOC.
3) Comparison (C): Use non-BMOC products (normal saline or sterile water) or blank control.
4) Outcome (O): Clinical treatment related indicators of children, including VAP, MVT, LOS, NEC, late-onset sepsis, mortality during hospitalization, time of full intestinal feeding, and time of full oral feeding.
5) Study design (S): Randomized controlled trial (RCT).

#### Exclusion Criteria

1) Preterm infants undergoing surgery.
2) The experimental group adopted BMOC combined with other intervention measures.
3) Retrospective studies, reviews, systematic evaluations, case reports, letters, reviews, or editorials.
4) Unable to obtain complete data.

### Data Extraction

Ambiguities about data extraction were resolved after discussion and consulting a third reviewer when necessary. The data extracted included: (1) Study title and country; (2) The first author and year of publication; (3) Research design; (4) Sample size; (5) Gestational age and birth weight; (6) MV baseline; (7) Method, time and frequency of intervention; (8) Outcomes: VAP, MVT, LOS, NEC, late-onset sepsis, mortality during hospitalization, time of full intestinal feeding, and time of full oral feeding.

### Quality Assessment

The quality of the included studies was evaluated and cross-checked by two researchers, respectively, according to the Cochrane Risk of Bias Tool (12), and consulted by a third reviewer when necessary. There are seven bias risk items. Make a judgment of “low risk,” “high risk,” and “unclear risk,” for each criterion, respectively. The included studies were rated as low bias risk if all of their bias risk items were rated as “low risk.” The included studies were rated as unclear bias risk if their bias risk items were rated as “low risk” and “risk unclear,” or all were rated as “unclear risk.” The included studies were rated as high bias risk if all of their bias risk items were rated as “high risk.”

### Statistical Analysis

RevMan 5.3 software was used for statistical analysis, and *P* < 0.05 was considered to be statistically significant. Statistical data such as the incidence of VAP and NEC, mortality during hospitalization, and late-onset sepsis used relative risk (RR) and 95% confidence intervals (CI) as effect statistics, while quantitative data such as MVT, LOS, time of full intestinal feeding, and time of full oral feeding used selective mean difference (MD) and 95% CI as effect statistics.

The heterogeneity among the included studies was represented by the *I*^2^ value. If the heterogeneity among the included studies was not significant (*I*^2^ < 50%, *P* > 0.1), the fixed-effect model was used to calculate the combined statistics. If there was significant heterogeneity among studies (*I*^2^ ≥ 50%, *P* ≤ 0.1), the random-effect model was used for analysis. Sensitivity analysis was performed by changing the effect model and using a one-by-one elimination method to evaluate the stability and reliability of the results. In addition, publication bias was detected by funnel plot.

## Results

### Literature Search

**Figure 1:**
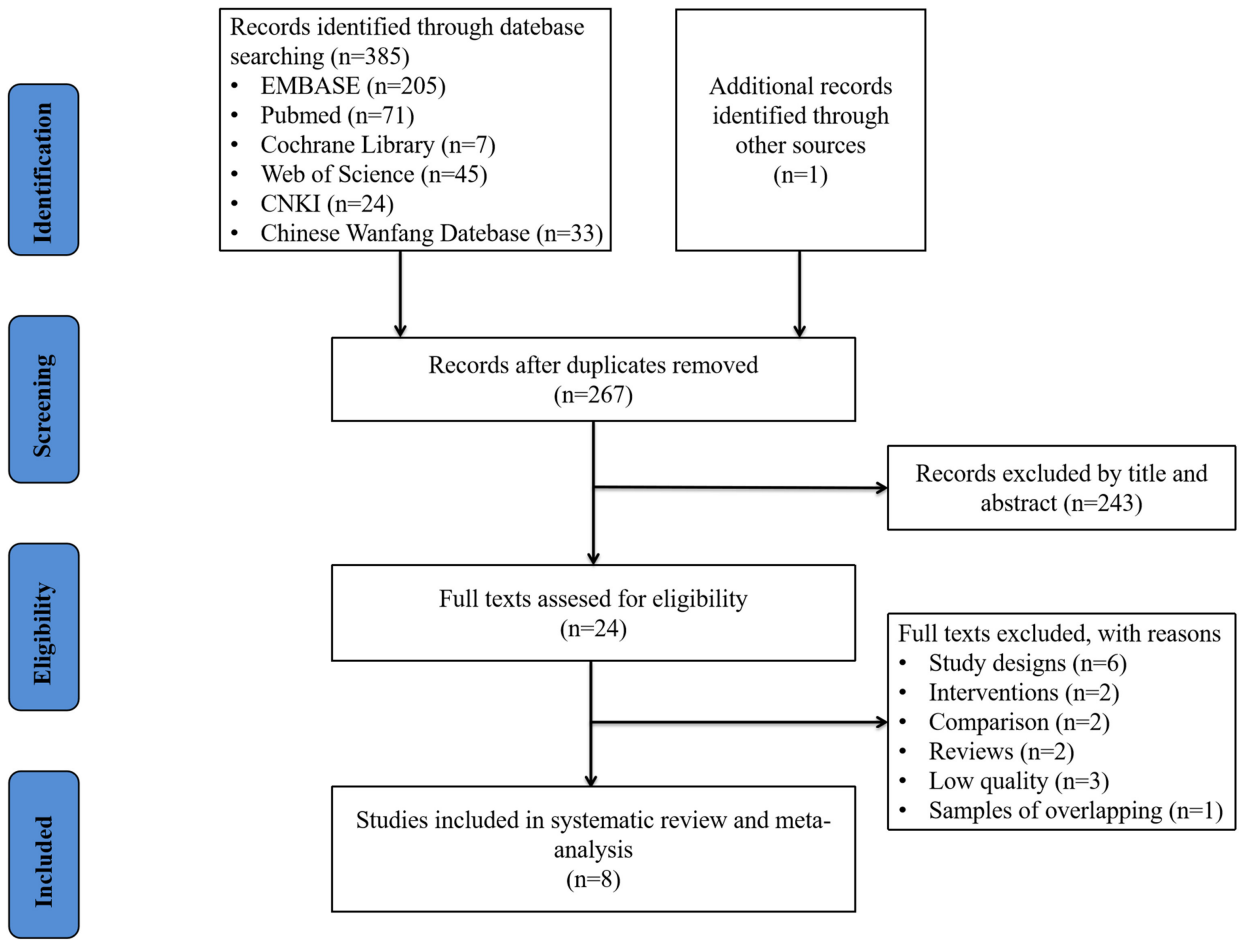
Literature screening process and results.

A database search identified 385 studies and a manual search included 1 study (13). One hundred and nineteen duplicated studies were excluded, and a total of 267 studies needed preliminary screening. After deleting obviously irrelevant literature by reading the title and abstract, the remaining 24 studies needed to be read in full. After reading the full text, 8 studies were finally identified for inclusion in this review (9, 10, 13–18). The process and results of literature screening are shown in Figure 1.

### Quality Assessment

**Figure 2:**
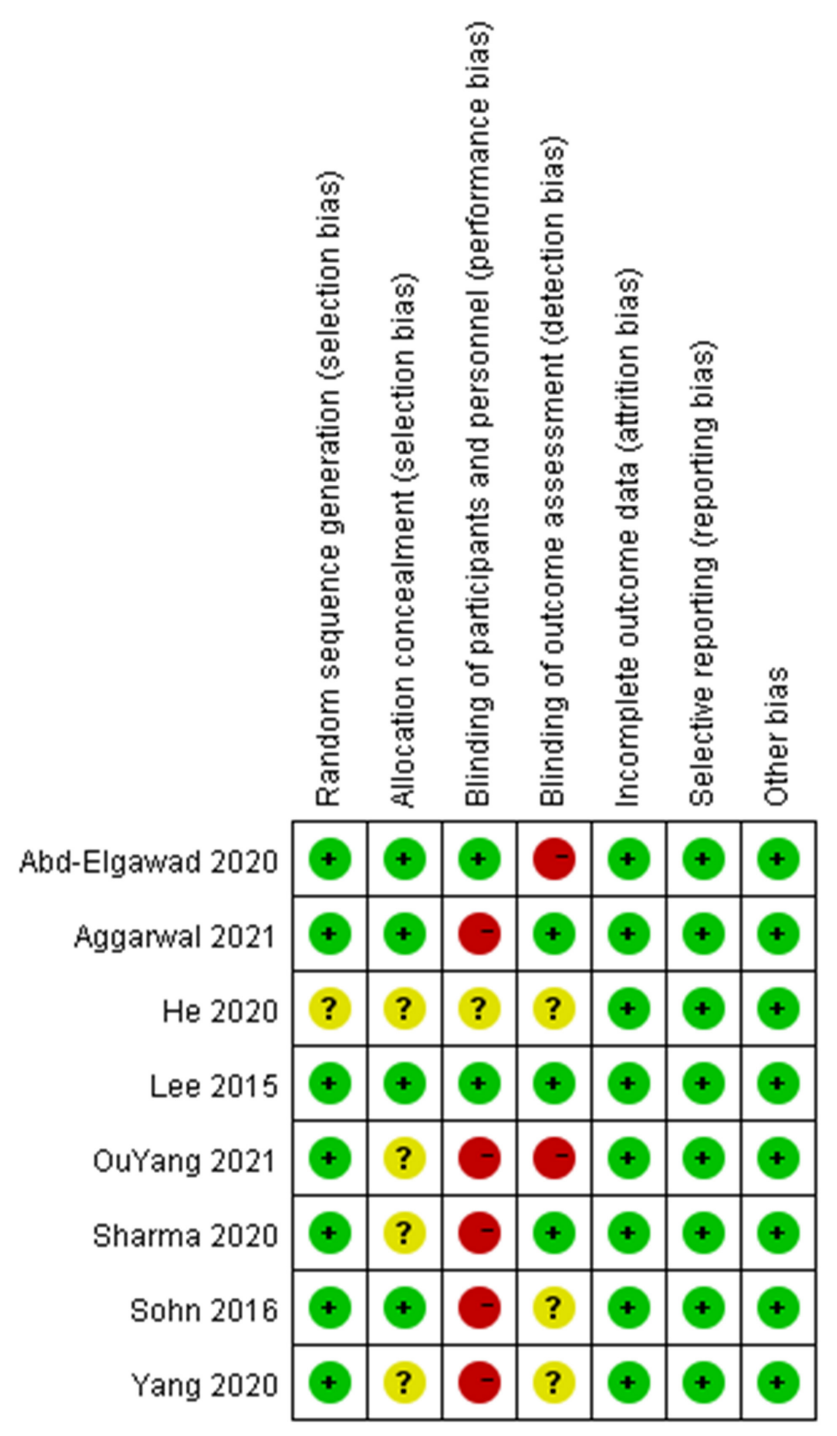
Authors' judgments about each risk of bias item for each included study.

**Figure 3:**
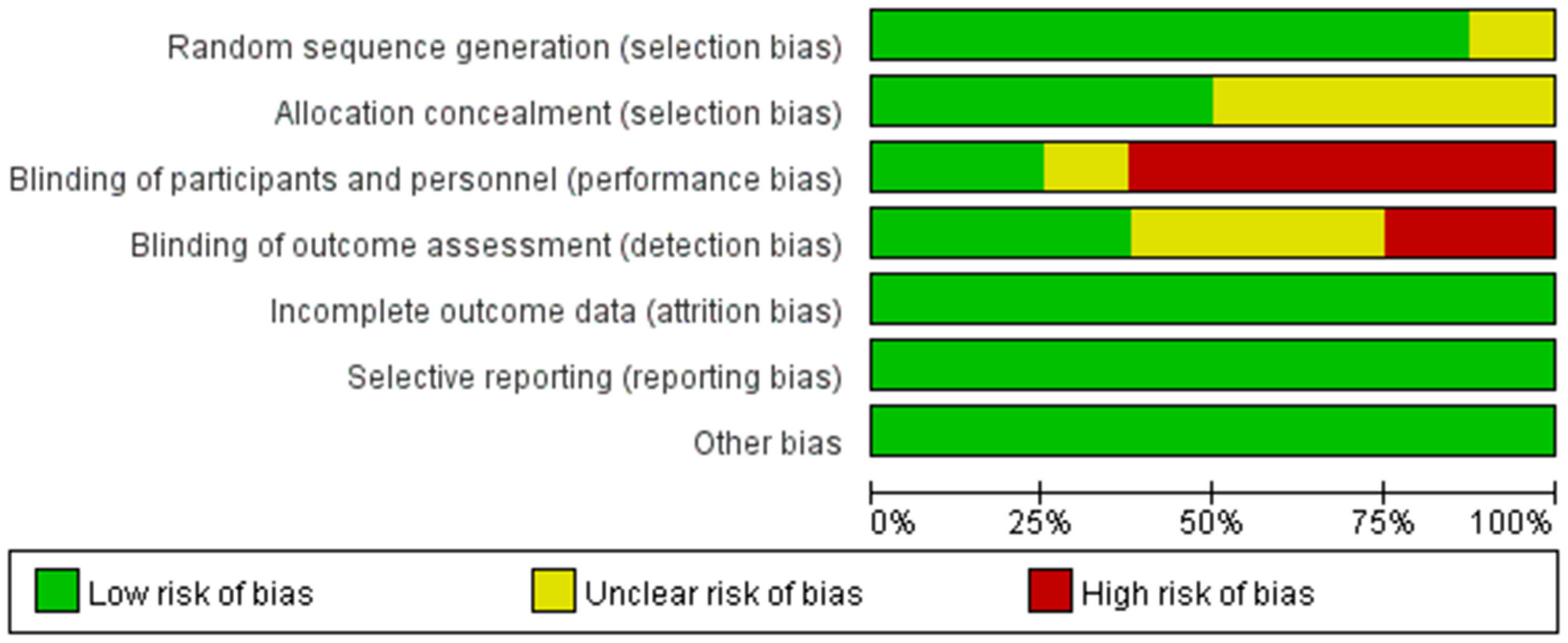
Authors' judgments about each risk of bias item presented as percentages across all included studies.

The risk of bias for all included studies is shown in Figures 2, 3. Among the 8 included studies, 1 study was evaluated as “low risk of bias” (13), 1 as “unclear risk of bias” (18), and 6 as “high risk of bias” (9, 10, 14–17).

### Study Characteristics

The study characteristics are shown in Table 1. The study covered the period from 2015 to 2021. Sample sizes ranged from 12 to 260 (1,046 in total). Among the 8 included studies, 6 were published in English (9, 10, 13–16), and 2 in Chinese (17, 18); 3 studies were conducted in China (16–18), 2 in India (9, 14), 1 in Egypt (10), 1 in South Korea (13), and 1 in the United States (15). Six studies used breast milk drop as an intervention (9, 10, 13–16), and two studies used breast milk scrub as an intervention (17, 18). The frequency of interventions ranged from every 2–8 h per day. There was no significant difference in gestational age and birth weight among the study groups (*P* > 0.05), which were comparable in baseline characteristics.

**Table 1:** Characteristics of the studies included in the meta-analysis.

**Study/Location**	**Study design**	**Risk of bias**	**Sample size**		**GA (W, M ±** ***SD*** **)**			**Birth weight (g, M ±** ***SD*** **)**			**MV baseline**			**Oral care solution**	**Dosage/Intervening measure/Interval time/Start Time/Time of duration**
			**EG**	**CG**	**EG**	**CG**	***P***	**EG**	**CG**	***P***	**EG**	**CG**	***P***		
Sharma et al. (9) India	RCT	High	59	58	29.1 ± 1.8	29.2 ± 1.9	0.78	1,146 ± 58	1,158 ± 61	0.76	Invasive (27.1%)	Invasive (31%)	0.70	EG: Colostrum CG: Blank control	0.2 ml/Drop/Every 2 h/Start after 24 h of postnatal life/Last 72 h
Abd-Elgawad et al. (10) Egypt	RCT	High	100	100	28.9 ± 2.05	28.8 ± 2.26	0.64	1,050 ± 246	1,022 ± 249	0.37	CV (32%) HFV (8%) Nasal CPAP (48%)	CV (37%) HFV (8%) Nasal CPAP (41%)	0.55 1.0 0.39	EG: Colostrum CG: Blank control	0.2 ml/Drop/Every 2–4 h/NA/Until the infants reached full oral feeding
Lee et al. (13) Korea	RCT	Low	24	24	26.7 ± 2.01	26.7 ± 2.43	>0.05	815 ± 291	830 ± 216	>0.05	Invasive (100%)	Invasive (100%)	>0.05	EG: Colostrum CG: Sterile distilled water	0.2 ml/Drop/Every 3 h/Begin at 48 h to 96 h after birth/Last 72 h
Aggarwal et al. (14) India	RCT	High	130	130	30 ± 2.22	30 ± 1.48	>0.05	1,205 ± 297	1,198 ± 259	>0.05	Invasive (40%)	Invasive (40%)	>0.05	EG: Colostrum CG: Sterile water	0.2 ml/Drop/Every 3 h/Begin within 24 h after birth/Until oral feeds were initiated
Sohn et al. (15) USA	RCT	High	6	6	27 ± 3.7	27 ± 2.2	>0.05	1,092 ± 637	1,015 ± 419	>0.05	Invasive (100%)	Invasive (100%)	>0.05	EG: Colostrum CG: Usual care	0.2 ml/Drop/Every 2 h/NA/Last 46 h
OuYang et al. (16) China	RCT	High	127	125	30.00 ± 1.83	29.65 ± 2.04	0.15	1,302 ± 210	1,329 ± 222	0.33	Invasive (NA) Non-invasive (NA)	Invasive (NA) Non-invasive (NA)	NA	EG: Colostrum CG: Normal saline	0.4 ml/Drop/Every 3 h/Start within 48 h after birth/Last for a total of 10 days
Yang et al. (17) China	RCT	High	50	50	34.5 ± 2.3	34.6 ± 2.1	>0.05	2,500 ± 300	2,500 ± 300	>0.05	Invasive (44%) Non-invasive (26%)	Invasive (46%) Non-invasive (24%)	>0.05 >0.05	EG: Breast milk CG: Normal saline	NA/Scrub/Every 8 h/NA/Until oral feeds were initiated
He et al. (18) China	RCT	Unclear	28	29	30.73 ± 1.84	30.77 ± 2.00	0.932	1,551 ± 439	1,611 ± 552	0.651	Invasive (100%)	Invasive (100%)	NA	EG: Colostrum CG: Normal saline	0.1 ml/Scrub/Every 4 h/NA/NA

*EG, experimental group; CG, control group; GA, gestational age; RCT, Randomized Control Trial; MV, mechanical ventilation; CV, conventional ventilation; HFV, high frequency ventilation; NA, No Application; W, week; h, hour; ml, milliliter; g, gram*.

### Meta-Analysis Results

#### The Incidence of VAP

**Figure 4:**
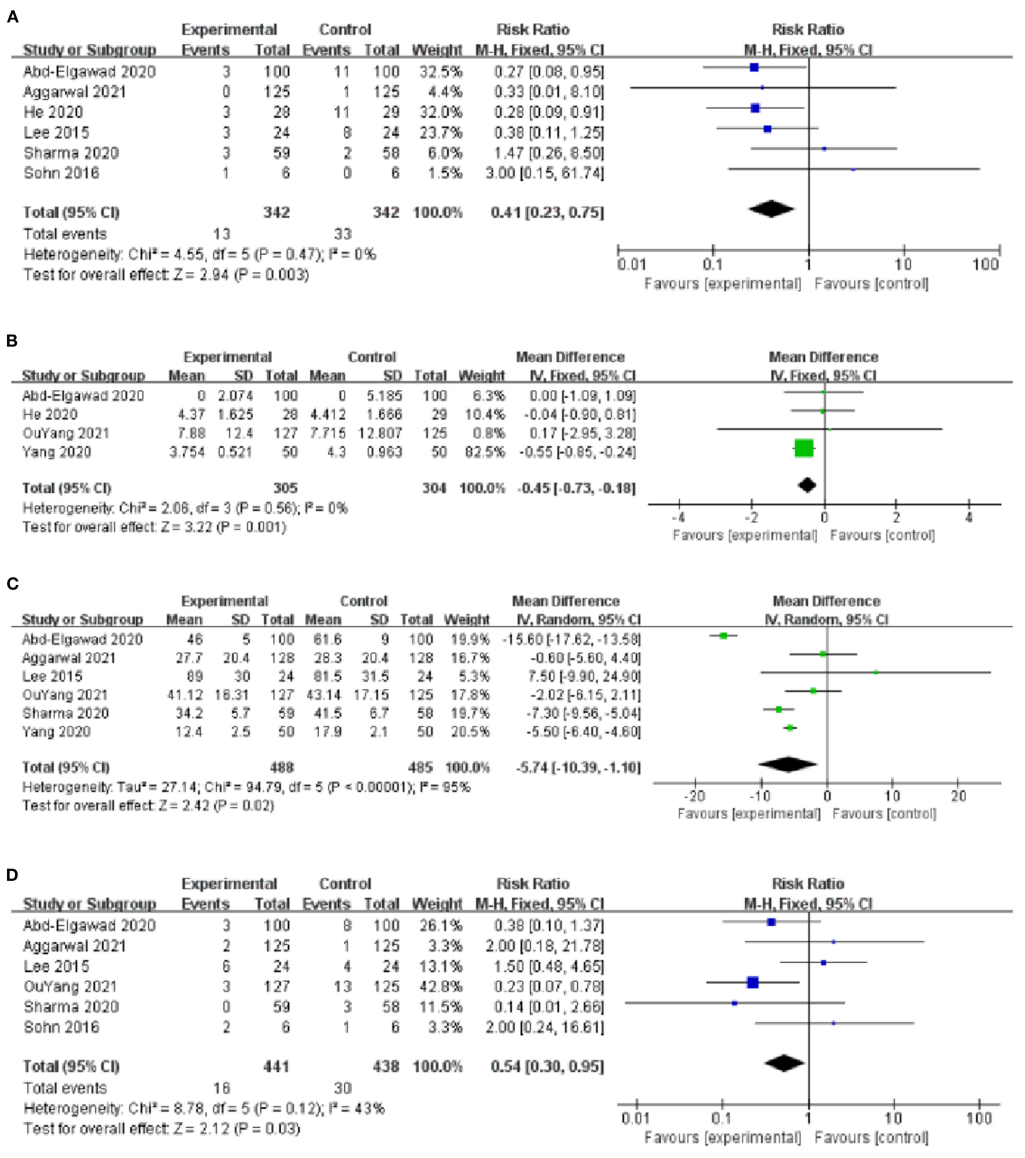
**(A)** Forest plot of the incidence of VAP. **(B)** Forest plot of the MVT. **(C)** Forest plot of the LOS. **(D)** Forest plot of the incidence of NEC.

The incidence of VAP was reported in 6 studies (9, 10, 13–15, 18), with low heterogeneity among studies (*I*^2^ = 0%, *P* = 0.47) and using a fixed-effect model analysis. The results showed that the incidence of VAP in the BMOC intervention group was significantly lower than in the control group [*RR* = 0.41, 95% *CI* (0.23, 0.75), *P* = 0.003] (Figure 4A). By changing the effect model for sensitivity analysis, the results did not change significantly, suggesting that the results were relatively stable [*RR* = 0.41, 95% *CI* (0.22, 0.77), *P* = 0.005].

#### Mechanical Ventilation Time

Four studies provided data on MVT (10, 16–18), with low heterogeneity among studies (*I*^2^ = 0%, *P* = 0.56) and using a fixed-effect model analysis. The results showed that the MVT of the BMOC intervention group was shorter than the control group [*MD* = −0.45, 95% *CI* (−0.73, −0.18), *P* = 0.001] (Figure 4B). By changing the effect model for sensitivity analysis, the results did not change, suggesting that the results were relatively stable [*MD* = −0.45, 95% *CI* (−0.73, −0.18), *P* = 0.001].

#### Length of Stay

The LOS was reported in 6 studies (9, 10, 13, 14, 16, 17), with significant heterogeneity among studies (*I*^2^ = 95%, *P* < 0.001) and using a random-effect model analysis. The results showed that the LOS of BMOC intervention group was shorter than the control group [*MD* = −5.74, 95% *CI* (−10.39, −1.10), *P* = 0.02] (Figure 4C). Sensitivity analysis was performed by using the one-by-one elimination method. After excluding the study of Abd-Elgawad et al. (10), the combined results did not change significantly [*MD* = −4.49, 95% *CI* (−6.83, −2.16), *P* < 0.001], but the heterogeneity decreased (*I*^2^ = 64%, *P* = 0.03), indicating that this study may be the source of heterogeneity (Table 2).

**Table 2:** The sensitivity analysis results of LOS, NEC, late onset sepsis and time of full intestinal feeding.

**Outcome**	**Before sensitivity analysis**			**References**	**After sensitivity analysis**		
	**Effect estimate**	***P***	***I*^2^ (%)**		**Effect estimate**	***P***	***I*^2^ (%)**
LOS	−5.74 (−10.39, −1.10)	0.02	95	Abd-Elgawad et al. (10)	−4.49 (−6.83, −2.16)	<0.001	64
NEC	0.54 (0.30, 0.95)	0.03	43	Lee et al. (13)	0.39 (0.20, 0.79)	0.009	24
Late onset sepsis	0.79 (0.40, 1.59)	0.51	51	OuYang et al. (16)	1.10 (0.67, 1.79)	0.71	0
Time of full intestinal feeding	−2.42 (−5.37, 0.52)	0.11	95	Abd-Elgawad et al. (10)	−1.61 (−4.14, 0.92)	0.21	79

*LOS, length of stay; NEC, necrotizing enterocolitis*.

#### The Incidence of NEC

Six studies provided data on the incidence of NEC (9, 10, 13–16), with moderate heterogeneity among studies (*I*^2^ = 43%, *P* = 0.12) and using a fixed-effect model analysis. The results showed that the incidence of NEC in the BMOC intervention group was lower than in the control group [*RR* = 0.54, 95% *CI* (0.30, 0.95), *P* = 0.03] (Figure 4D). Sensitivity analysis was performed by using the one-by-one elimination method. After excluding the study of Lee et al. (13), the combined results did not change significantly [*RR* = 0.39, 95% *CI* (0.20, 0.79), *P* = 0.009], but the heterogeneity decreased (*I*^2^ = 24%, *P* = 0.26), indicating that this study may be the source of heterogeneity (Table 2).

#### Mortality During Hospitalization

**Figure 5:**
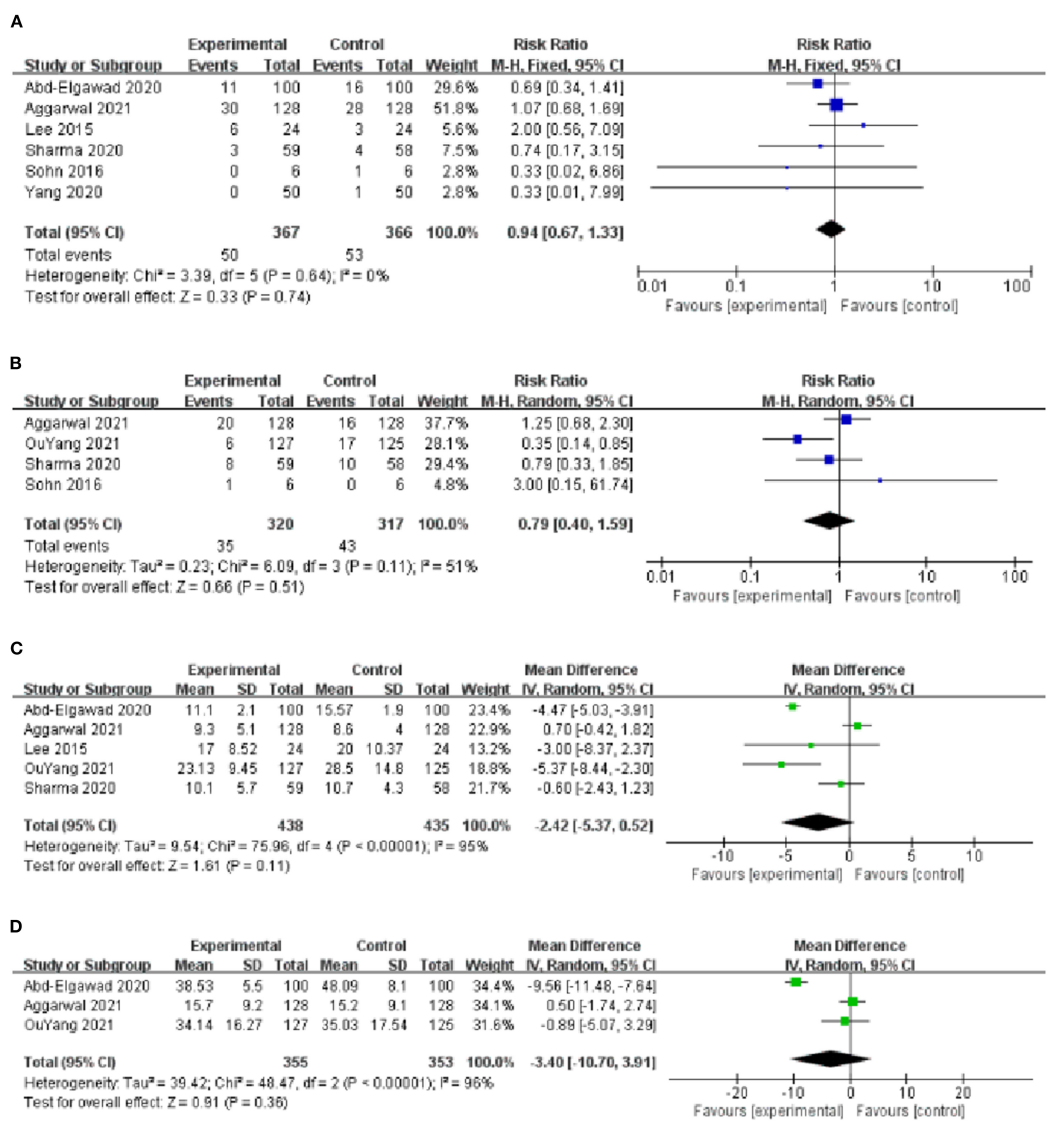
**(A)** Forest plot of the mortality during hospitalization. **(B)** Forest plot of the late-onset sepsis. **(C)** Forest plot of the time of full intestinal feeding. **(D)** Forest plot of the time of full oral feeding.

Six studies evaluated the effect of BMOC on the mortality during hospitalization (9, 10, 13–15, 17), with low heterogeneity among studies (*I*^2^ = 0%, *P* = 0.64), and the fixed-effect model analysis was used to consolidate the effect value. The results showed that there was no significant difference in mortality during hospitalization between the two groups [*RR* = 0.94, 95% *CI* (0.67, 1.33), *P* = 0.74] (Figure 5A). By changing the effect model for sensitivity analysis, the results did not change significantly [*RR* = 0.93, 95% *CI* (0.61, 1.44), *P* = 0.76], suggesting that the results were relatively stable.

#### Late-Onset Sepsis

Four studies reported the effect of BMOC on late-onset sepsis (9, 14–16), with moderate heterogeneity among studies (*I*^2^ = 51%, *P* = 0.11) and using a random-effect model analysis. The results showed that there was no significant difference in late-onset sepsis between the two groups [*RR* = 0.79, 95% *CI* (0.40, 1.59), *P* = 0.51] (Figure 5B). Sensitivity analysis was performed by using the one-by-one elimination method. After excluding the study of Yang et al. (16), the combined results did not change significantly [*RR* = 1.10, 95% *CI* (0.67, 1.79), *P* = 0.71], but the heterogeneity decreased (*I*^2^ = 0%, *P* = 0.55), indicating that this study may be the source of heterogeneity (Table 2).

#### Time of Full Intestinal Feeding

Five studies reported the effect of BMOC on the time of full intestinal feeding (9, 10, 13, 14, 16), with significant heterogeneity among studies (*I*^2^ = 95%, *P* < 0.001) and using a random effect model analysis. The results showed that there was no significant difference in the time of full intestinal feeding between the two groups [*MD* = −2.42, 95% *CI* (−5.37, 0.52), *P* = 0.11] (Figure 5C). Sensitivity analysis was performed by using the one-by-one elimination method. After excluding the study of Abd-Elgawad et al. (10), the combined results did not change significantly [*MD* = −1.61, 95% *CI* (−4.14, 0.92), *P* = 0.21], but the heterogeneity decreased (*I*^2^ = 79%, *P* = 0.002), indicating that this study may be the source of heterogeneity (Table 2).

#### Time of Full Oral Feeding

Three studies reported the effect of BMOC on the time of full oral feeding (10, 14, 16), with significant heterogeneity among studies (*I*^2^ = 96%, *P* < 0.001) and using a random-effect model analysis. The results showed that there was no significant difference in the time of full oral feeding between the two groups [*MD* = −3.40, 95% *CI* (−10.70, 3.91), *P* = 0.36] (Figure 5D).

### Publication Bias

**Figure 6:**
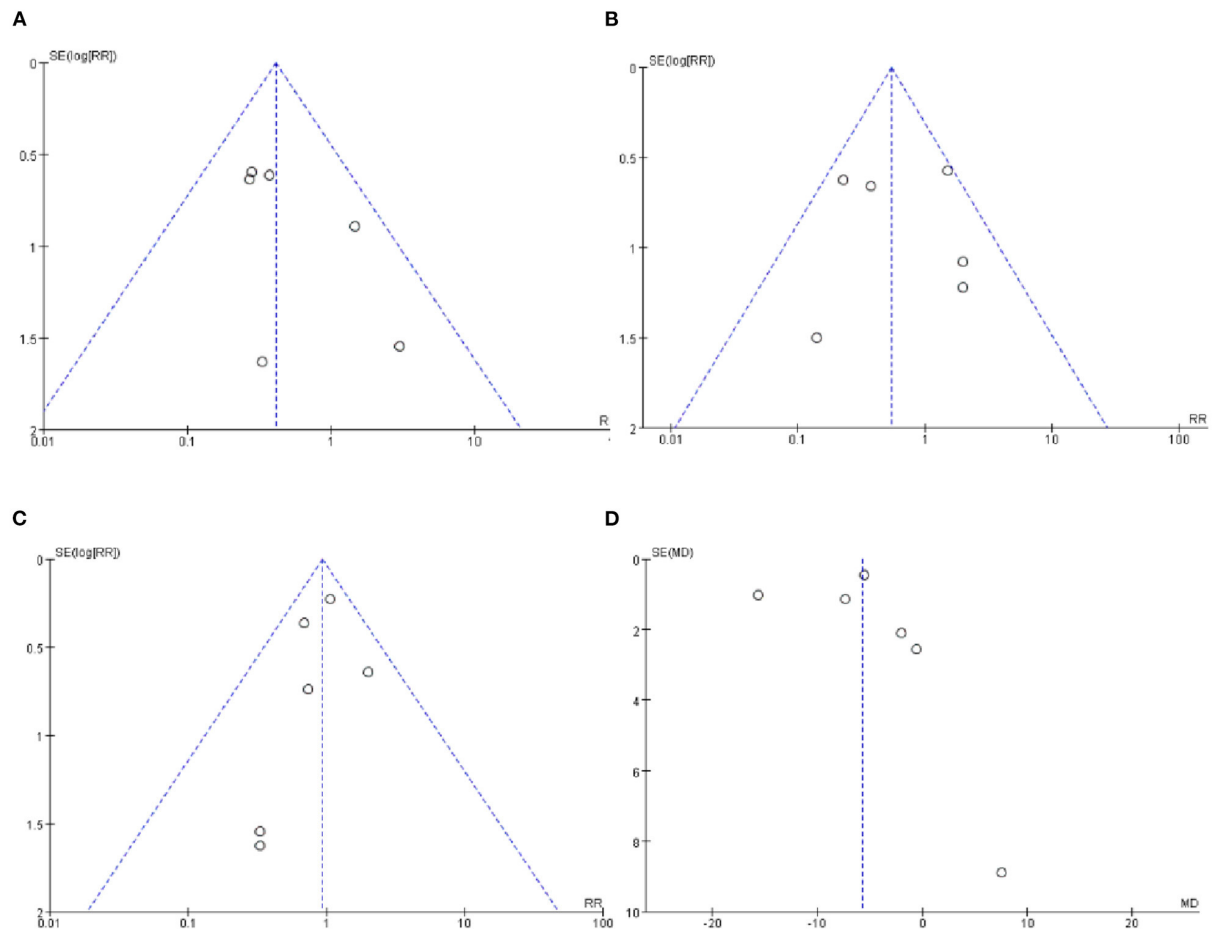
**(A)** Funnel plot of the incidence of VAP. **(B)** Funnel plot of the incidence of NEC. **(C)** Funnel plot of the mortality during hospitalization. **(D)** Funnel plot of the LOS.

The funnel plots of VAP, NEC and the mortality during hospitalization were visually symmetrical and did not show a significant risk of publication bias (Figures 6A–C). The funnel plot of LOS was visually asymmetric, suggesting the possibility of publication bias (Figure 6D).

## Discussion

In recent years, BMOC is reported to be beneficial to infant health, which is safe, feasible, and cost effective (19). This systematic review and meta-analysis identified 8 studies involving 1,046 preterm infants, and the results showed that BMOC had a positive effect on reducing the incidence of VAP and NEC and shortening MVT and LOS for mechanically ventilated preterm infants, however, no effect was observed on late-onset sepsis, mortality during hospitalization, time of full intestinal feeding and time of full oral feeding between the two groups.

VAP is one of the most common hospital infections in the neonatal intensive care unit, and the main reason is that the artificial airway destroys the natural mechanical barrier of oral and nasal mucosa against pathogens, which provides a direct and rapid way for oropharyngeal colonization bacteria to enter the lower respiratory tract (4). Once VAP occurs in mechanically ventilated preterm infants, it is extremely easy to cause difficulty in weaning, prolong MVT and LOS, and increase hospitalization cost and mortality (20–22). Studies have shown that poor oral hygiene or dry mouth is one of the common factors affecting bacterial colonization, and effective oral care can prevent the colonization of pathogenic microorganisms in the upper respiratory tract and reduce the incidence of VAP (23). At present, the commonly used oral care solutions in clinical practice are normal saline (24), chlorhexidine (25) and sodium bicarbonate (26), etc., but all of them have various deficiencies. Research have shown that secretory IgA (sIgA), lactoferrin, and other active components in breast milk can be absorbed through oropharyngeal mucosa, activate the infant immune system, and kill pathogenic microorganisms such as streptococcus pneumonia and chlamydia spores. At the same time, it can form a protective layer on the oral surface of preterm infants, block bacterial colonization of mucosa and play a first-line defense role (27, 28). Oral care with breast milk can exert the immune effect of breast milk and reduce the incidence of VAP by reducing oropharyngeal and endotracheal pathogens in children with MV (29), which is consistent with the results of this study. As an objective indicator, VAP has small heterogeneity and high credibility and therefore highlights the usefulness of BMOC to prevent the occurrence of VAP among mechanically ventilated preterm infants.

The results of this study showed that BMOC can shorten MVT and LOS, which is closely related to the reduction of VAP. This result is consistent with the view of Li et al. (30), who found that MVT and LOS in patients without VAP infection were significantly lower than in those with VAP infection. Breast milk is the most natural, safest, and completely natural food for the growth of infants (31). It is the best choice for newborns, especially for preterm infants. It can not only provide nutrients such as amino acids but also enhance their resistance to pathogenic microorganisms, which is conducive to the rehabilitation of mechanically ventilated newborns.

We also found that BMOC can reduce the incidence of NEC. NEC is the most common and serious cause of gastrointestinal-related incidence rate and mortality in preterm infants (32). Probiotics such as bifidobacteria in breast milk can help preterm infants establish intestinal flora, improve the capacity of the intestinal immune system, and protect intestinal mucosa from excessive stimulation (33). A recent study had shown that sIgA in breast milk could prevent the development of NEC in preterm infants (34), Rodriguez et al. (27) inhaled colostrum into the oral cavity of preterm infants through syringes, and found sIgA and lactoferrin in their urine and tracheal aspirates, suggesting that breast milk could be absorbed by mucosa, which could reasonably explain our results.

However, no significant difference in the mortality during hospitalization was observed between the two groups. The reason may be that the mortality of preterm infants was affected by many factors, such as gestational age, weight, infectious diseases, etc. (35). In our study, we observed no significant difference in late-onset sepsis between the two groups, and the reason may be that the stimulation effect of breast milk on lactoferrin was not persistent (36). The existing evidence in this study cannot prove that BMOC has an impact on the time to reach full intestinal feeding and full oral feeding, which may be related to the different gestational age or birth weight of preterm infants, resulting in the difference of enteral feeding tolerance (37). In addition, there are differences in feeding strategies implemented in different hospitals, which may also affect the results (38).

## Limitations

Although we adjusted the study population from mechanically ventilated infants to mechanically ventilated preterm infants to improve the homogeneity and comparability of the study, there are still some limitations in this systematic review and meta-analysis. First, only 6 databases were retrieved in this meta-analysis, and the language was limited to Chinese and English, so there may be incomplete retrieval. Second, there was heterogeneity, due to differences between studies in the implementation of BMOC, such as intervention methods (including drop and scrub), intervention start time, intervention end time, intervention frequency and dose. However, we were unable to conduct subgroup analysis due to the small number of included studies. It is suggested that clinical medical staff should pay attention to the effects of different BMOC time, methods, frequency, and doses on mechanically ventilated preterm infants, to seek the best intervention scheme. Third, the funnel plot of LOS suggested a possible risk of publication bias, so caution should be taken in interpreting the results. In the future, large sample and high-quality RCTs are still needed to further verify the safety and effectiveness of BMOC.

## Conclusion

According to the summary analysis of the currently available data, the use of BMOC is helpful to reduce the incidence of VAP and NEC, and shorten MVT and LOS in mechanically ventilated preterm infants. The results should be treated cautiously as the differences in the intervention schemes of different studies. In the future, it is still necessary to carry out large sample and high-quality RCT studies to further clarify the application effect of BMOC on mechanically ventilated preterm infants.

## Data Availability Statement

The original contributions presented in the study are included in the article/Supplementary Material. Further inquiries can be directed to the corresponding author.

## Author Contributions

MC and YL: research design. MC: later stages of the design and writing of this paper. MC and LL: identifying and screening of the included randomized controlled trials. MC and YP: data analysis and evaluation. YL and LC: manuscript revision. All authors suggestions to the data analysis, helping in interpretation of results, and final manuscript reading and approval.

## Funding

This study was supported by the Fujian Provincial Finance Special Project (2020CZ011), the Guiding Project of Fujian Science and Technology (2021Y0023), and Fujian Key Laboratory of Cardio-Thoracic Surgery (Fujian Medical University).

## Conflict of Interest

The authors declare that the research was conducted in the absence of any commercial or financial relationships that could be construed as a potential conflict of interest.

## Publisher's Note

All claims expressed in this article are solely those of the authors and do not necessarily represent those of their affiliated organizations, or those of the publisher, the editors and the reviewers. Any product that may be evaluated in this article, or claim that may be made by its manufacturer, is not guaranteed or endorsed by the publisher.
