# Supplementary material for: Effect of Breast Milk Oral Care on Mechanically Ventilated Preterm Infants: A Systematic Review and Meta-Analysis of Randomized Controlled Trials
Source: Front Pediatr. 2022 Jul 7;10:899193. doi: 10.3389/fped.2022.899193 (PMC9301042; doi:10.3389/fped.2022.899193)
Supplement: Supplementary file 2 [file Data_Sheet_2.DOC]

**File S2. Search strategy**

**Embase (n=205)**

#1: 'colostrum'/exp

#2: 'breast milk'/exp

#3: colostrum*:ab,ti

#4: human OR breast OR expressed OR mother* OR maternal OR donor*

#5: milk*

#6: #4 AND #5

#7: #1 OR #2 OR #3 OR #6

#8: 'negative pressure ventilator'/exp

#9: 'endotracheal intubation'/exp

#10: 'mechanical ventilator'/exp

#11: intubat*:ab,ti OR ventilat*:ab,ti OR respirator*:ab,ti

#12: artificial AND near AND respirat* OR (invasive AND near AND ventil*)

#13: #8 OR #9 OR #10 OR #11 OR #12

#14: 'child'/exp

#15: 'newborn'/exp

#16: 'prematurity'/exp

#17: child*:ab,ti OR infant*:ab,ti OR neonat*:ab,ti OR newborn:ab,ti OR paediatric*:ab,ti OR toddler:ab,ti

#18: #14 OR #15 OR #16 OR #17

#19: #7 AND #13 AND #18

#20: 'mouth hygiene'/exp

#21: oral AND care OR (mouth AND care) OR (oral AND hygien*) OR 'oral hygien*' OR (dental AND hygien*) OR orophary*

#22: #20 OR #21

#23: #19 AND #22

**PubMed (n=71)**

#1: ("Colostrum"[Mesh]) OR (Colostrum*[Title/Abstract])

#2: "Milk, Human"[Mesh]

#3: ((((((human) OR (breast)) OR (expressed)) OR (mother*)) OR (maternal)) OR (donor*)) AND (milk*)

#4: #1 OR #2 OR #3

#5: "Intubation, Intratracheal"[Mesh]

#6: (intubat* near ?tracheal) OR (intubat*[Title/Abstract])

#7: "Ventilators, Negative-Pressure"[Mesh]

#8: "Ventilators, Mechanical"[Mesh]

#9: ventilat*[Title/Abstract]

#10: respirator*[Title/Abstract]

#11: (artificial near respirat*) OR (invasive near ventil*)

#12: #5 OR #6 OR #7 OR #8 OR #9 OR #10 OR #11

#13: #4 AND #12

#14: "Child"[Mesh]

#15: "Infant, Newborn"[Mesh]

#16: "Infant, Premature"[Mesh]

#17: (((((child*[Title/Abstract]) OR (infant*[Title/Abstract])) OR (neonat*[Title/Abstract])) OR (newborn[Title/Abstract])) OR (paediatric*[Title/Abstract])) OR (toddler[Title/Abstract])

#18: (((((Infant*, Premature[Title/Abstract]) OR (Premature Infant*[Title/Abstract])) OR (Preterm Infant*[Title/Abstract])) OR (Infants, Preterm[Title/Abstract])) OR (Neonatal Prematurity[Title/Abstract])) OR (Prematurity, Neonatal[Title/Abstract])

#19: #14 OR #15 OR #16 OR #17 OR #18

#20: #13 AND #19

#21: "Oral Hygiene"[Mesh]

#22: (((((oral care) OR (mouth care)) OR (oral hygien*)) OR (oral-hygien*)) OR (dental hygien*)) OR (orophary*)

#23: #21 OR #22

#24: #20 AND #23

**Cochrane Library（n=7）**

#1: MeSH descriptor: [Colostrum] explode all trees

#2: MeSH descriptor: [Milk, Human] explode all trees

#3: ("Colostrum*")

#4: ("human") OR ("breast") OR ("expressed")

#5: ("mother*") OR ("maternal") OR ("donor*")

#6: ("milk*")

#7: #1 OR #2

#8: #4 OR #5

#9: #6 AND #8

#10: #3 OR #7

#11: #9 OR #10

#12: MeSH descriptor: [Ventilators, Negative-Pressure] explode all trees

#13: MeSH descriptor: [Intubation, Intratracheal] explode all trees

#14: MeSH descriptor: [Ventilators, Mechanical] explode all trees

#15: ("intubat* near ?tracheal") OR ("intubat*")

#16: ("ventilat*") OR ("respirator*")

#17: ("artificial near respirat*") OR ("invasive near ventil*")

#18: #12 OR #13

#19: #14 OR #15

#20: #16 OR #17

#21: #18 OR #19

#22: #20 OR #21

#23: MeSH descriptor: [Child] explode all trees

#24: MeSH descriptor: [Infant, Newborn] explode all trees

#25: MeSH descriptor: [Infant, Premature] explode all trees

#26: ("child*") OR ("infant*") OR ("neonat*")

#27: ("newborn") OR ("paediatric*") OR ("toddler")

#28: #23 OR #24

#29: #25 OR #26

#30: #28 OR #29

#31: #27 OR #30

#32: #11 AND #22

#33: #31 AND #32

#34: MeSH descriptor: [Oral Hygiene] explode all trees

#35: ("oral care") OR ("mouth care") OR ("oral hygien*") OR ("oral-hygien*") OR ("dental hygien*")

#36: #34 OR #35

#37: #33 AND #36

**Web of Science (n=45)**

#1: colostrum* (All Fields)

#2: human (All Fields) or breast (All Fields) or expressed (All Fields) or mother* (All Fields) or maternal (All Fields) or donor* (All Fields)

#3: milk* (All Fields)

#4: (#2) AND #3

#5: (#1) OR #4

#6: intubat* (All Fields) or ventilat* (All Fields) or respirator* (All Fields)

#7: child* (All Fields) or infant* (All Fields) or neonat* (All Fields) or newborn (All Fields) or paediatric* (All Fields) or toddler (All Fields) or prematur* (All Fields)

#8: ((#5) AND #6) AND #7

#9: oral care (All Fields) or mouth care (All Fields) or oral hygien* (All Fields) or oral-hygien* (All Fields) or dental hygien* (All Fields) or orophary* (All Fields)

#10: #8 AND #9

**Chinese Database (n=57)**

1. 母乳[主题]
2. 初乳[主题]
3. #1 OR #2
4. 机械通气[主题]
5. 气管插管[主题]
6. 呼吸机[主题]
7. OR #4-6
8. #3 AND #7
9. 婴儿[主题]
10. 新生儿[主题]
11. 早产儿[主题]
12. 儿童[主题]
13. OR #9-12
14. #8 AND #13
15. 口腔[主题]
16. #14 AND #15
